# Supplementary figures and images for: A de novo assembly of the sweet cherry (Prunus avium cv. Tieton) genome using linked-read sequencing technology
Source: PeerJ. 2020 Jun 5;8:e9114. doi: 10.7717/peerj.9114 (PMC7278891; doi:10.7717/peerj.9114)

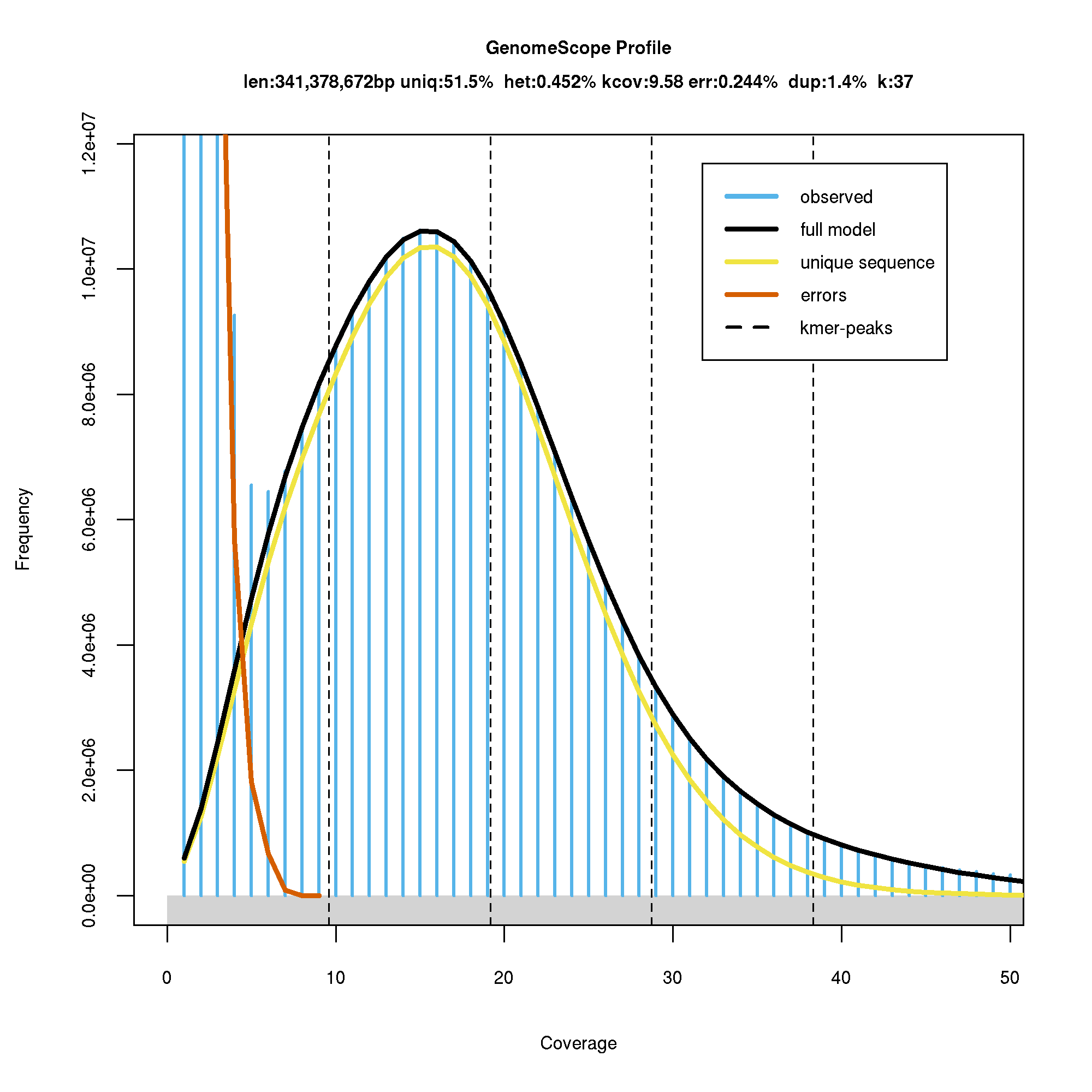

Supplement: Supplemental Information 1 [file peerj-08-9114-s001.png]
