# Supplementary material for: A de novo assembly of the sweet cherry (Prunus avium cv. Tieton) genome using linked-read sequencing technology
Source: PeerJ. 2020 Jun 5;8:e9114. doi: 10.7717/peerj.9114 (PMC7278891; doi:10.7717/peerj.9114)
Supplement: Supplemental Information 2 [file peerj-08-9114-s002.docx]

**Table S1.** Genome annotations used for gene orthologous analysis in this study

| **Species** | **Annotation version** | **Reference** |
| --- | --- | --- |
| *A.thaliana* | TAIR10 | ([Sloan et al. 2018](#_ENREF_6)) |
| *C.sinensis* | Version 1.0 | ([Xu et al. 2013](#_ENREF_11)) |
| *F.vesca* | Version 2.0a2 | ([Li et al. 2017](#_ENREF_4)) |
| *M.domestica* | Version 1.1 | ([Daccord et al. 2017](#_ENREF_3)) |
| *P.mume* | Version 1.0 | ([Zhang et al. 2012](#_ENREF_12)) |
| *P.persica* | Version 2.0 | ([Verde et al. 2017](#_ENREF_9)) |
| *P.yedoensis* | Version 1.0 | ([Baek et al. 2018](#_ENREF_1)) |
| *P.bretschneideri* | Version 1.1 | ([Wu et al. 2013](#_ENREF_10)) |
| *R.chinensis* | Version 2.0 | ([Raymond et al. 2018](#_ENREF_5)) |
| *R.occidentalis* | Version 3.0 | ([VanBuren et al. 2018](#_ENREF_8)) |
| *S.lycopersicum* | Version 3.0 | ([Tomato Genome 2012](#_ENREF_7)) |
| *V.vinifera* | VCost.v3 | ([Canaguier et al. 2017](#_ENREF_2)) |

**References:**

Baek S, Choi K, Kim GB, Yu HJ, Cho A, Jang H, Kim C, Kim HJ, Chang KS, Kim JH, and Mun JH. 2018. Draft genome sequence of wild Prunus yedoensis reveals massive inter-specific hybridization between sympatric flowering cherries. *Genome Biology* 19:127. 10.1186/s13059-018-1497-y

Canaguier A, Grimplet J, Di Gaspero G, Scalabrin S, Duchene E, Choisne N, Mohellibi N, Guichard C, Rombauts S, Le Clainche I, Berard A, Chauveau A, Bounon R, Rustenholz C, Morgante M, Le Paslier MC, Brunel D, and Adam-Blondon AF. 2017. A new version of the grapevine reference genome assembly (12X.v2) and of its annotation (VCost.v3). *Genom Data* 14:56-62. 10.1016/j.gdata.2017.09.002

Daccord N, Celton JM, Linsmith G, Becker C, Choisne N, Schijlen E, van de Geest H, Bianco L, Micheletti D, Velasco R, Di Pierro EA, Gouzy J, Rees DJG, Guerif P, Muranty H, Durel CE, Laurens F, Lespinasse Y, Gaillard S, Aubourg S, Quesneville H, Weigel D, van de Weg E, Troggio M, and Bucher E. 2017. High-quality de novo assembly of the apple genome and methylome dynamics of early fruit development. *Nature Genetics* 49:1099-1106. 10.1038/ng.3886

Li Y, Wei W, Feng J, Luo H, Pi M, Liu Z, and Kang C. 2017. Genome re-annotation of the wild strawberry Fragaria vesca using extensive Illumina- and SMRT-based RNA-seq datasets. *DNA Research*. 10.1093/dnares/dsx038

Raymond O, Gouzy J, Just J, Badouin H, Verdenaud M, Lemainque A, Vergne P, Moja S, Choisne N, Pont C, Carrere S, Caissard JC, Couloux A, Cottret L, Aury JM, Szecsi J, Latrasse D, Madoui MA, Francois L, Fu X, Yang SH, Dubois A, Piola F, Larrieu A, Perez M, Labadie K, Perrier L, Govetto B, Labrousse Y, Villand P, Bardoux C, Boltz V, Lopez-Roques C, Heitzler P, Vernoux T, Vandenbussche M, Quesneville H, Boualem A, Bendahmane A, Liu C, Le Bris M, Salse J, Baudino S, Benhamed M, Wincker P, and Bendahmane M. 2018. The Rosa genome provides new insights into the domestication of modern roses. *Nature Genetics* 50:772-777. 10.1038/s41588-018-0110-3

Sloan DB, Wu Z, and Sharbrough J. 2018. Correction of Persistent Errors in Arabidopsis Reference Mitochondrial Genomes. *Plant Cell* 30:525-527. 10.1105/tpc.18.00024

Tomato Genome C. 2012. The tomato genome sequence provides insights into fleshy fruit evolution. *Nature* 485:635-641. 10.1038/nature11119

VanBuren R, Wai CM, Colle M, Wang J, Sullivan S, Bushakra JM, Liachko I, Vining KJ, Dossett M, Finn CE, Jibran R, Chagne D, Childs K, Edger PP, Mockler TC, and Bassil NV. 2018. A near complete, chromosome-scale assembly of the black raspberry (Rubus occidentalis) genome. *Gigascience* 7. 10.1093/gigascience/giy094

Verde I, Jenkins J, Dondini L, Micali S, Pagliarani G, Vendramin E, Paris R, Aramini V, Gazza L, Rossini L, Bassi D, Troggio M, Shu S, Grimwood J, Tartarini S, Dettori MT, and Schmutz J. 2017. The Peach v2.0 release: high-resolution linkage mapping and deep resequencing improve chromosome-scale assembly and contiguity. *BMC Genomics* 18:225. 10.1186/s12864-017-3606-9

Wu J, Wang Z, Shi Z, Zhang S, Ming R, Zhu S, Khan MA, Tao S, Korban SS, Wang H, Chen NJ, Nishio T, Xu X, Cong L, Qi K, Huang X, Wang Y, Zhao X, Wu J, Deng C, Gou C, Zhou W, Yin H, Qin G, Sha Y, Tao Y, Chen H, Yang Y, Song Y, Zhan D, Wang J, Li L, Dai M, Gu C, Wang Y, Shi D, Wang X, Zhang H, Zeng L, Zheng D, Wang C, Chen M, Wang G, Xie L, Sovero V, Sha S, Huang W, Zhang S, Zhang M, Sun J, Xu L, Li Y, Liu X, Li Q, Shen J, Wang J, Paull RE, Bennetzen JL, Wang J, and Zhang S. 2013. The genome of the pear (Pyrus bretschneideri Rehd.). *Genome Research* 23:396-408. 10.1101/gr.144311.112

Xu Q, Chen LL, Ruan X, Chen D, Zhu A, Chen C, Bertrand D, Jiao WB, Hao BH, Lyon MP, Chen J, Gao S, Xing F, Lan H, Chang JW, Ge X, Lei Y, Hu Q, Miao Y, Wang L, Xiao S, Biswas MK, Zeng W, Guo F, Cao H, Yang X, Xu XW, Cheng YJ, Xu J, Liu JH, Luo OJ, Tang Z, Guo WW, Kuang H, Zhang HY, Roose ML, Nagarajan N, Deng XX, and Ruan Y. 2013. The draft genome of sweet orange (Citrus sinensis). *Nature Genetics* 45:59-66. 10.1038/ng.2472

Zhang Q, Chen W, Sun L, Zhao F, Huang B, Yang W, Tao Y, Wang J, Yuan Z, Fan G, Xing Z, Han C, Pan H, Zhong X, Shi W, Liang X, Du D, Sun F, Xu Z, Hao R, Lv T, Lv Y, Zheng Z, Sun M, Luo L, Cai M, Gao Y, Wang J, Yin Y, Xu X, Cheng T, and Wang J. 2012. The genome of Prunus mume. *Nat Commun* 3:1318. 10.1038/ncomms2290
