# Supplementary material for: A de novo assembly of the sweet cherry (Prunus avium cv. Tieton) genome using linked-read sequencing technology
Source: PeerJ. 2020 Jun 5;8:e9114. doi: 10.7717/peerj.9114 (PMC7278891; doi:10.7717/peerj.9114)
Supplement: Supplemental Information 3 [file peerj-08-9114-s003.docx]

**Table S2.** Statistics of RNA sequencing of sweet cherry (*Prunus avium*) cv. Tieton.

| **RNA sequencing feature** | **Value** |
| --- | --- |
| **Raw reads number** | 78,982,458 |
| **Raw base pairs** | 11,844,153,498 |
| **Clean reads number** | 77,258,972 |
| **Clean base pairs** | 11,585,524,087 |
| **Clean rate (%)** | 97.82 |
| **Q20 (%)** | 97.65 |
| **Q30 (%)** | 93.03 |
