# Supplementary material for: A de novo assembly of the sweet cherry (Prunus avium cv. Tieton) genome using linked-read sequencing technology
Source: PeerJ. 2020 Jun 5;8:e9114. doi: 10.7717/peerj.9114 (PMC7278891; doi:10.7717/peerj.9114)
Supplement: Supplemental Information 4 [file peerj-08-9114-s004.docx]

**Table S3.** Statistics of sweet cherry (*Prunus avium*) cv. Tieton genome assembly using Supernova v2.0 with 40x, 50x, 60x, 65x, 68x, 70x, and 75x coverage of linked reads.

|  | **Coverage** | | | | | | |
| --- | --- | --- | --- | --- | --- | --- | --- |
| **Assembly parameter** | **40x** | **50x** | **60x** | **65x** | **68x** | **70x** | **75x** |
| **Reads used (Million)** | 90 | 113 | 136 | 147.01 | 154.01 | 158.01 | 169.01 |
| **contig N50 (kb)** | 50.47 | 55.16 | 54.14 | 52.5 | 53.36 | 51.01 | 50.83 |
| **scaffold N50 (Mb)** | 0.79 | 1.64 | 2.19 | 3.21 | 3.21 | 3.45 | 2.69 |

The contig scaffold N50 were calculated based on scaffold sequences >=10kb.
