# Supplementary material for: A de novo assembly of the sweet cherry (Prunus avium cv. Tieton) genome using linked-read sequencing technology
Source: PeerJ. 2020 Jun 5;8:e9114. doi: 10.7717/peerj.9114 (PMC7278891; doi:10.7717/peerj.9114)
Supplement: Supplemental Information 5 [file peerj-08-9114-s005.docx]

**Table S4.** Summary of 150 Million reads mapping against sweet cherry (*Prunus avium*) cv. Tieton genome assembly using Burrows-Wheller Alignment (BWA) tool.

| **Assembly parameter** | **Value** |
| --- | --- |
| **Total reads (Million)** | 150 |
| **Total mapped reads (Million)** | 148.83 |
| **Total mapping rate** | 99.02% |
| **Genome coverage rate (1x)** | 99.99% |
| **Genome coverage rate (10x)** | 99.35% |
| **Genome coverage rate (30x)** | 86.15% |
| **Genome coverage rate (50x)** | 44.50% |
