# Supplementary material for: A de novo assembly of the sweet cherry (Prunus avium cv. Tieton) genome using linked-read sequencing technology
Source: PeerJ. 2020 Jun 5;8:e9114. doi: 10.7717/peerj.9114 (PMC7278891; doi:10.7717/peerj.9114)
Supplement: Supplemental Information 6 [file peerj-08-9114-s006.docx]

**Table S5.** Summary of sweet cherry (*Prunus avium*) cv. Tieton genome completeness assessed by Core Eukaryotic Genes Mapping Approach (CEGMA) and Benchmarking Universal Single-Copy Orthologs (BUSCO).

|  | **CEGMA** | |
| --- | --- | --- |
|  | **Number** | **Percent (%)** |
| **Total genes** | 248 | 100 |
| **Complete genes** | 231 | 93.15 |
| **Partial genes** | 13 | 5.24 |
| **Missing genes** | 4 | 1.61 |
|  | **BUSCO** | |
| **Total genes** | 1440 | 100 |
| **Complete and single-copy genes** | 1345 | 93.4 |
| **Complete and duplicated genes** | 36 | 2.56 |
| **Fragmented genes** | 22 | 1.52 |
| **Missing genes** | 37 | 2.57 |
