# Supplementary material for: A de novo assembly of the sweet cherry (Prunus avium cv. Tieton) genome using linked-read sequencing technology
Source: PeerJ. 2020 Jun 5;8:e9114. doi: 10.7717/peerj.9114 (PMC7278891; doi:10.7717/peerj.9114)
Supplement: Supplemental Information 7 [file peerj-08-9114-s007.docx]

**Table S6.** Summary of scaffolds anchored to pseudo-chromosomes of sweet cherry (*Prunus avium*) cv. Tieton genome.

|  | **Anchored** | **Oriented** | **Unplaced** |
| --- | --- | --- | --- |
| **Markers (unique)** | 7,838 | 7,266 | 308 |
| **Markers per Mb** | 36.6 | 35.9 | 4.7 |
| **N50 Scaffolds** | 22 | 25 | 0 |
| **Scaffolds** | 494 | 688 | 13,850 |
| **Scaffolds with 1 marker** | 288 | 0 | 223 |
| **Scaffolds with 2 markers** | 76 | 10 | 28 |
| **Scaffolds with 3 markers** | 19 | 6 | 4 |
| **Scaffolds with 4 markers** | 111 | 97 | 3 |
| **Total base pair (bp)** | 214,271,005 | 202,585,018 | 66,056,220 |
| **Percent of total assembly** | 76.4% | 72.3% | 23.6% |
