# Supplementary material for: A de novo assembly of the sweet cherry (Prunus avium cv. Tieton) genome using linked-read sequencing technology
Source: PeerJ. 2020 Jun 5;8:e9114. doi: 10.7717/peerj.9114 (PMC7278891; doi:10.7717/peerj.9114)
Supplement: Supplemental Information 8 [file peerj-08-9114-s008.docx]

**Table S7.** Length distributions of the RNA-seq assembly of sweet cherry (*Prunus avium*) cv. Tieton using Trinity.

| **Length (bp)** | **Total number** | **Percentage (%)** |
| --- | --- | --- |
| **200-300** | 5,832 | 17.46 |
| **300-500** | 4,894 | 14.65 |
| **500-1000** | 6,417 | 19.21 |
| **1000-2000** | 9,167 | 27.45 |
| **2000-5000** | 6,753 | 20.22 |
| **> 5000** | 338 | 1.01 |
| **Total number** | 33,401 | - |
| **Total length** | 42,636,522 | - |
| **N40 length** | 2,292 | - |
| **N50 length** | 1,981 | - |
| **N60 length** | 1,699 | - |
| **N70 length** | 1,405 | - |
