# Supplementary material for: A de novo assembly of the sweet cherry (Prunus avium cv. Tieton) genome using linked-read sequencing technology
Source: PeerJ. 2020 Jun 5;8:e9114. doi: 10.7717/peerj.9114 (PMC7278891; doi:10.7717/peerj.9114)
Supplement: Supplemental Information 9 [file peerj-08-9114-s009.docx]

**Table S8.** Statistics of gene family analysis between sweet cherry (*Prunus avium*) cv. Tieton and the other 12 plant species.

|  | ***A.thaliana*** | ***C.sinensis*** | ***F.vesca*** | ***M.domestica*** | ***P.avium*** | ***P.mume*** | ***P.persica*** | ***P.yedoensis*** | ***P.bretschneideri*** | ***R.chinensis*** | ***R.occidentalis*** | ***S.lycopersicum*** | ***V.vinifera*** | **Total** |
| --- | --- | --- | --- | --- | --- | --- | --- | --- | --- | --- | --- | --- | --- | --- |
| **Number of genes** | 27,416 | 25,379 | 33,538 | 45,116 | 30,975 | 31,390 | 26,873 | 41,294 | 44,840 | 45,469 | 33,286 | 35,768 | 41,733 | 463,077 |
| **Number of genes in orthogroups** | 22,207 | 22,244 | 26,555 | 36,870 | 28,913 | 27,743 | 24,574 | 34,482 | 42,988 | 32,241 | 24,327 | 25,471 | 26,878 | 375,493 |
| **Number of unassigned genes** | 5,209 | 3,135 | 6,983 | 8,246 | 2,062 | 3,647 | 2,299 | 6,812 | 1,852 | 13,228 | 8,959 | 10,297 | 14,855 | 87,584 |
| **Percentage of genes in orthogroups** | 81 | 87.6 | 79.2 | 81.7 | 93.3 | 88.4 | 91.4 | 83.5 | 95.9 | 70.9 | 73.1 | 71.2 | 64.4 | 81.1 |
| **Percentage of unassigned genes** | 19 | 12.4 | 20.8 | 18.3 | 6.7 | 11.6 | 8.6 | 16.5 | 4.1 | 29.1 | 26.9 | 28.8 | 35.6 | 17.3 |
| **Number of orthogroups containing species** | 13,051 | 14,457 | 15,899 | 16,389 | 15,904 | 16,764 | 16,160 | 16,553 | 14,299 | 17,150 | 15,525 | 14,237 | 15,358 | 23,129 |
| **Percentage of orthogroups containing species** | 56.4 | 62.5 | 68.7 | 70.9 | 68.8 | 72.5 | 69.9 | 71.6 | 61.8 | 74.1 | 67.1 | 61.6 | 66.4 | 100 |
| **Number of species-specific orthogroups** | 82 | 53 | 54 | 14 | 46 | 28 | 4 | 31 | 10 | 23 | 27 | 76 | 77 | 525 |
| **Number of genes in species-Specific orthogroups** | 707 | 261 | 317 | 60 | 124 | 177 | 12 | 99 | 33 | 66 | 118 | 484 | 462 | 2920 |
| **Percentage of genes in species-specific orthogroups** | 2.6 | 1 | 0.9 | 0.1 | 0.4 | 0.6 | 0 | 0.2 | 0.1 | 0.1 | 0.4 | 1.4 | 1.1 | 0.6 |
| **Number of orthogroups with all species presented** | 8,465 | | | | | | | | | | | | | |
| **Number of single-copy orthogroups** | 246 | | | | | | | | | | | | | |
